# Hippocampal–caudate nucleus interactions support exceptional memory performance

**DOI:** 10.1007/s00429-017-1556-2

**Published:** 2017-11-14

**Authors:** Nils C. J. Müller, Boris N. Konrad, Nils Kohn, Monica Muñoz-López, Michael Czisch, Guillén Fernández, Martin Dresler

**Affiliations:** 1 Donders Institute for Brain, Cognition and Behaviour, Radboud University Medical Centre, Nijmegen, The Netherlands; 2 Max Planck Institute of Psychiatry, Munich, Germany; 3 Human Neuroanatomy Laboratory, School of Medicine and Regional Centre for Biomedical Research, University of Castilla-La Mancha, Albacete, Spain

**Keywords:** Memory athletes, Method of loci, Stimulus response learning, Cognitive map, Hippocampus, Caudate nucleus

## Abstract

Participants of the annual World Memory Championships regularly demonstrate extraordinary memory feats, such as memorising the order of 52 playing cards in 20 s or 1000 binary digits in 5 min. On a cognitive level, memory athletes use well-known mnemonic strategies, such as the method of loci. However, whether these feats are enabled solely through the use of mnemonic strategies or whether they benefit additionally from optimised neural circuits is still not fully clarified. Investigating 23 leading memory athletes, we found volumes of their right hippocampus and caudate nucleus were stronger correlated with each other compared to matched controls; both these volumes positively correlated with their position in the memory sports world ranking. Furthermore, we observed larger volumes of the right anterior hippocampus in athletes. Complementing these structural findings, on a functional level, fMRI resting state connectivity of the anterior hippocampus to both the posterior hippocampus and caudate nucleus predicted the athletes rank. While a competitive interaction between hippocampus and caudate nucleus is often observed in normal memory function, our findings suggest that a hippocampal–caudate nucleus cooperation may enable exceptional memory performance. We speculate that this cooperation reflects an integration of the two memory systems at issue-enabling optimal combination of stimulus-response learning and map-based learning when using mnemonic strategies as for example the method of loci.

**Electronic supplementary material:**

The online version of this article (10.1007/s00429-017-1556-2) contains supplementary material, which is available to authorized users.

## Introduction

People differ in their ability to memorise information. However, participants of memory championships—memory athletes—exhibit a completely different scale of memory performance. They are able to memorise more information quicker and more reliably than what is within the normal range of memory performance: Remembering 300 random words in only 15 min without a single mistake is not a feat one can just perform. However, the memory athletes tested here are capable of this and similar feats. One central pillar of their success is a mnemonic strategy that is known for its encoding efficacy since ancient Greece: the method of loci (Roediger 1980; Yates 1966). Users of this strategy mentally navigate a familiar route and at separate loci—distinct landmarks along the route—visualise placing the information there. This combination of map-based spatial memory and associative memory has repeatedly been demonstrated to enhance memory for a broad variety of information (Worthen and Hunt 2011).

Successful memory athletes attribute their memory performance mainly to the method of loci (Dresler et al. 2017; Dresler and Konrad 2013; Maguire et al. 2003). Little is known, however, why the method of loci facilitates memory retention so strongly. One explanation might be that the method engages different memory systems synergistically. In the classification of memory subsystems two aspects are often contrasted (Squire 2004): habits or simple stimulus–response association (Jog et al. 1999; Knowlton et al. 1996; Mishkin and Petri 1984; Yin and Knowlton 2006) and more episodic and map-like representations (Eichenbaum 2004; O’Keefe and Nadel 1978). While the former is linked to the caudate nucleus, the latter is linked to the hippocampus. This division is exemplified in the context of navigation: a stimulus response strategy would rely on simple association of landmarks and actions (“turn right at the church”). In contrast, navigation using the map-based system would rely on an internal map of the environment. As efficiency of these two systems depends on the environmental context, they often compete for the task at hand so the ideal system for the task is utilised (Doeller et al. 2008; Poldrack and Packard 2003).

During the method of loci, new information needs to be associated with the loci; and after successful encoding of one piece of information, one needs to navigate to the next locus as quickly as possible (Mallow et al. 2015). For the association (Knowlton et al. 1996; Yin and Knowlton 2006) and the automatic navigation along a well-known, fixed route—characteristically for stimulus–response learning—(Hartley et al. 2003; Packard and Knowlton 2002) the caudate nucleus appears ideally suited. Memory athletes routinely create a vivid visual image for the association of new information on a given locus. For the vividness (Danker et al. 2016), for constructing a visual scene (Hassabis and Maguire 2011; Zeidman and Maguire 2016), and for maintaining a map of the whole set of information along the route (O’Keefe and Nadel 1978) the hippocampus is usually recruited. The routes memory athletes use are ones that they are extremely familiar with containing many different loci. For this kind of representations, the hippocampus with its map-based encoding should be ideal (O’Keefe and Nadel 1978). During training of the method of loci, the memory athletes would train over and over again to use these routes. Memory athletes tend to train multiple routes so that during a competition they do not need to reuse the same route, which potentially might lead to interference. At a competition, the well-rehearsed routes are then used to encode novel information, going along the route. Taken together, on the one hand the rapid navigation from locus to locus would be served well with efficient stimulus–response associations provided by the caudate nucleus. Whereas on the other hand, the vivid scene construction needed for encoding and the maintaining of a global representation of the route could be done by the hippocampus. Integrating these facilities in a frictionless fashion might be what enables memory athletes’ superior memory. Preliminary evidence about the involvement of the caudate nucleus and the hippocampus comes from work on mnemonics: Both the method of loci and the pegword method, a similarly associative but non-spatial mnemonic, show caudate nucleus activity during encoding, however, only the method of loci elicits increased hippocampal activation (Fellner et al. 2016). This supports the specific involvement of the hippocampus in the spatial dimension of the method of loci.

There is substantial evidence for a competitive interaction of the hippocampus and the caudate nucleus: during spatial navigation, lesioning the one system improved performance based on the other system and vice versa (Poldrack and Packard 2003). This double dissociation implies that when both systems are intact, they are competing for the task at hand, which in turn reduces their efficiency (Lee et al. 2008; Packard et al. 1989). However, using early stage Huntington disease as a model for lesions in the caudate nucleus, a compensatory role of the hippocampal system has been observed; while the function of the caudate nucleus decays, the hippocampus can rescue the loss of functionality. Furthermore, in the same study, they observed a cooperative interaction of the memory systems in healthy controls which facilitated route recognition performance (Voermans et al. 2004). We hypothesise a similar cooperative interaction between the hippocampus and the caudate nucleus in memory athletes to facilitate their memory performance as it supports the method of loci optimally.

We investigated 23 athletes out of the Top-50 of the memory sports world ranking and 23 controls matched for age, sex, and IQ. To study whether memory athletes show a stronger synergy between the hippocampus and the caudate nucleus, we combined structural analysis and functional analysis of resting-state brain connectivity. We are not comparing task activation of memory athletes to matched controls as that is confounded by performance differences. Therefore, it is difficult to distinguish whether observed differences in activation are cause or consequence of behavioural differences.

In contrast to matched controls, athletes might exhibit more refined mechanisms for mnemonic processing or utilise a qualitatively different approach in terms of neural processing. To capture both of these differences, our analysis strategy is twofold: comparing our sample to matched controls, we test how they differ structurally and functionally; relating structural and functional variation within the athlete sample to their position in the world ranking, we investigate what predicts their success. Both analyses complement each other. The comparison to the control group can reveal anatomical changes common among the athletes, while the association to the world ranking can identify anatomical patterns that are central to the success of the athletes. As previous work showed a functional gradient along the anterior to posterior axis of the hippocampus (Strange et al. 2014) that is directly implicated in spatial processing (Kjelstrup et al. 2008), we subdivided the hippocampus into anterior and posterior part. The anterior and posterior hippocampus have been dissociated functionally on many aspects of cognition (Poppenk et al. 2013). A secondary reason for this was that an enlarged posterior hippocampus could be accompanied by a shrunken anterior hippocampus—producing no difference on average (Maguire et al. 2006).

We hypothesise that a specific trait or the massive training of the memory athlete is associated to structural differences in volumes of the hippocampus and the caudate nucleus; these should be accompanied by functional interactions that facilitate the synergistic use during the method of loci.

## Methods

### Sample

The mnemonic ability of the memory athletes is represented by their position in the international memory sports world rankings (IAM; http://www.iam-stats.com/). This ranking is based on a score that is calculated on the basis of their personal performance records in memory competitions that test ten memory events. We recruited 23 memory athletes (age: mean 27.8 years, range 19–51 years; 14 males) of the Top-50 (at the time of their participation 2010–2013) of the memory sports world rankings via email, phone calls or personally. All of these participants attribute their superior memory skills to deliberate training in mnemonic strategies. Control participants (age: mean 28.1 years, range 20–53 years; 14 males), were matched for age, sex, handedness, smoking status, and IQ. Where relevant, to ensure matching with the generally high intellectual level of the memory athletes, control participants were recruited among gifted students of academic foundations and members of the Mensa society (http://www.mensa.de) via mailing lists. All participants were paid and provided written informed consent to the study in line with the approval by the ethics committee of the Medical Faculty of the University of Munich.

### Procedure

The control participants performed a fluid reasoning test (Weiß and Weiß 2006) and a standardised memory test (Bäumler 1974) during a separate screening session. Furthermore, we checked for the following exclusion criteria: experience in mnemonic strategies, psychiatric or neurological history, and drug abuse. The memory test included six subtests assessing the learning and retention of figural, verbal, and numerical information. It was conducted to avoid including participants that are naturally exceptional memorisers. We planned to exclude participants with a performance of more than two standard deviations above the mean according to norms provided with the test (Bäumler 1974); however, none of the participants reached this criterion. The fluid reasoning test was used to match control participants to the memory athletes, thus preventing that differences in mnemonic abilities can be explained by differences in fluid reasoning. Most of the memory athletes already completed the fluid reasoning test for a separate earlier study, the remaining ones completed it after the MRI part. For all the control participants and athletes, we first acquired an anatomical scan followed by an 8 min resting state scan. As part of another study, 17 participants of both the control and athlete sample performed a word-encoding task followed by another resting state scan and a diffusion-weighted anatomical scan. Immediately after leaving the scanner, participants had to indicate on a 4-point scale if they had been continuously alert, partly tired, partly drowsy, or partly asleep during the rs-fMRI scan, and if they had their eyes closed during the resting state and open during the encoding session. Analysis of this data indicated that all participants adhered to the eyes closed instructions and no participant reported having been drowsy or asleep during rs-fMRI.

### MRI data acquisition and analysis

All imaging data were collected at the Max Planck Institute of Psychiatry, Munich, using a 3T (GE Discovery MR750) scanner with a 12-channel head coil. A standard localiser and a 3D T1-weighted anatomical scan (TR 7.1 ms, TE 2.2 ms, slice thickness 1.3 mm, in-plane FOV 240 mm, 320 × 320 × 128 matrix, 12° flip angle) preceded fMRI data collection. Eight minutes of resting state fMRI with eyes closed were collected (EPI sequence, TR 2.5 s, TE 30 ms, flip angle 90°), covering the whole brain with 34 slices, using a 64 × 64 matrix with 3 mm slice thickness and 1 mm slice spacing, and a field of view of 240 × 240 mm^2^. The images were AC–PC aligned and acquired using an interleaved slice acquisition scheme.

### Volumetric analysis

The anatomical images were bias field-corrected using N4 (Tustison et al. 2010). We then used the advanced normalisation toolbox (ANTs) (Avants et al. 2011a, b) to generate a study-specific template using an iterative procedure of diffeomorphic registrations including all structural scans (Avants and Gee 2004). This template was used as a reference for all further functional and structural registrations. This kind of template has been demonstrated to be especially useful for nonstandard populations that show hippocampal alterations (Avants et al. 2010). For the registration of the functional volumes, we resampled the template to an isotropic resolution of 2 mm.

For segmenting the hippocampus, we used a semiautomatic procedure. In a first step, a subset of hippocampi was manually segmented by a trained anatomist (MML), we then used this to train a multi-atlas segmentation algorithm with joint label fusion implemented in ANTs (Wang and Yushkevich 2013). This was then applied to automatically segment all hippocampi. To separate the hippocampi into anterior (head) and posterior part (body + tail), we manually identified the uncal apex as detailed in Weiss et al. (2005) and Poppenk et al. (2013) on the structural images. For segmenting the caudate nucleus, we used FSL first (Patenaude et al. 2011). Both segmentation algorithms utilised non-linear transformations and operated in each participants’ native space.

From the segmentations, volumes were extracted using FSL fslstats. All statistical analyses regarding the structural volumes were conducted using SPSS 21 (Armonk, NY: IBM Corp). To test for between group differences in volumes for the hippocampus and caudate nucleus, we used linear mixed models. The model for the hippocampus included the fixed factors group, anterior/posterior hippocampus, hemisphere, intracranial volume, age and gender, and a random intercept. The model for the caudate nucleus included the fixed factors group, hemisphere, intracranial volume, age and gender, and a random intercept. To correct for violations of sphericity due to the small sample size, we applied Greenhouse–Geisser correction to the *F* statistics. To test the association of the hippocampus and caudate nucleus volumes with the world ranking position, we used partial correlations controlling for differences in intracranial volume. Significance of these correlations was determined after correction for multiple comparisons applying Bonferroni correction to both the correlations of the hippocampus and the caudate nucleus. For the hippocampus, the level of significance that was used was *p* < 0.05/4 = 0.0125 and for the caudate nucleus, *p* < 0.05/2 = 0.025. Correlations were compared using Fisher *r*-to-*z* transformations (Steiger 1980). *p* values between 0.05 and 0.1 are referred to as trend; values below 0.05 indicate significance.

### Functional connectivity analysis

The resting state scans were preprocessed using FSL 5.0.8 (Jenkinson et al. 2012): we applied motion correction using MCFLIRT, slice-timing correction, spatial smoothing using a Gaussian kernel of FWHM 6 mm and normalisation of the entire 4D time series by a single multiplicative factor. The first two volumes were discarded to allow for the magnetisation to reach equilibrium. Next, we used ICA-AROMA to clean up the data from participant movement and other noise components using an independent component analysis approach (Pruim et al. 2015a, b). Afterwards, we extracted the mean time series from the white matter and cerebrospinal fluid compartments and regressed these. Individual white matter and cerebrospinal fluid masks for each participant were obtained using a six class segmentation on the anatomical scans (Avants et al. 2011). Finally, we applied a 100-s high pass filter to remove slow drifts. Registrations to the study-specific template were carried out using FLIRT to register the functional to the anatomical scans and FNIRT to register the anatomical scan to the study-specific template (Jenkinson et al. 2012). To generate functional masks for the regions of interest for the functional analysis, we registered the anatomical segmentations to the native functional space.

To assess the functional connectivity, we used a seed-based approach informed by the results of the volumetric analysis. In a first step for each subject, the first eigenvariate of the right anterior hippocampus was extracted. Using a general linear model this eigenvariate was then spatially regressed against the 4D time series resulting in one connectivity value per voxel. These connectivity images were then warped into group space using the above-mentioned transformations. In the group space, we calculated the statistics as described below. For 17 of the 23 athletes and for their respective pairs in the matched controls, we had a second resting state scan from the same scanner with identical parameters as they participated in an additional study. To increase reliability, we generated the connectivity map for both scans, when available, and combined them, using fixed effects before using them in the group analysis resulting in increased connectivity estimates.

All comparisons for the functional connectivity analysis were tested for statistical significance using nonparametric permutation testing implemented in FSL randomise. We used 10.000 permutation samples and threshold free cluster enhancement (TFCE) (Smith and Nichols 2009; Winkler et al. 2014). For the between-group comparison we used a two sample *t*-test, whereas for the testing the association of functional connectivity and the world ranking position, we used a centred parametric regressor of the scores from which the world ranking is derived. While the *t*-test reveals how athletes would differ from controls, the parametric regressor tests whether connectivity from the seed region to a different region correlate with the world ranking within the memory athlete sample. These results were then small-volume corrected using a mask comprising the right caudate nucleus as well as the right posterior hippocampus. For reporting, we warped the results in a final step into MNI152 space.

## Results

### Volumetric analysis

**Fig. 1:**
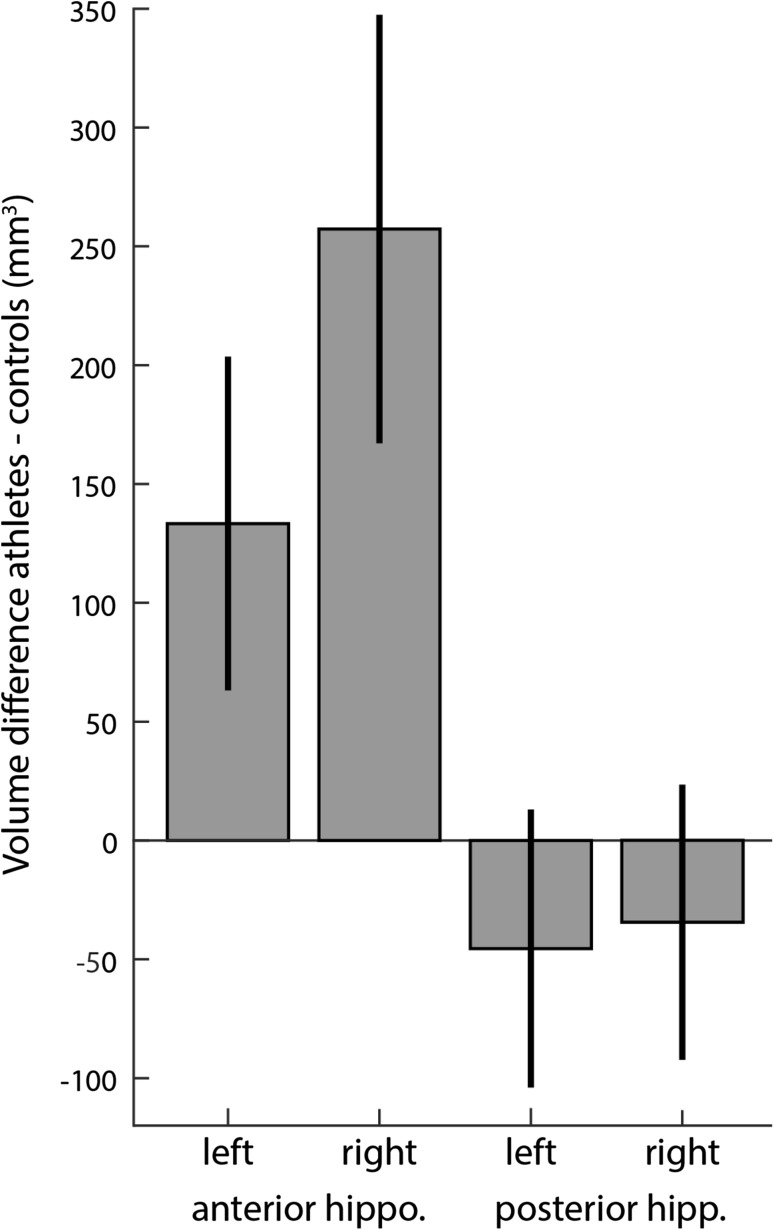
Hippocampal volume difference of memory athletes vs. matches controls. Comparing the volumes of the hippocampi using mixed models showed two significant interactions: group with hemisphere (*F*_(1,44)_ = 5, *p* = 0.03) and group with position (anterior vs. posterior, *F*_(1,44)_ = 5.41, *p* = 0.025). The right hippocampus of athletes is larger compared to controls (MD 111.42, *p* = 0.021). Furthermore the anterior part is also relatively enlarged in athletes (MD 195.33, *p* = 0.016). Together, these two two-way interactions lead to the biggest volumetric difference being in the right anterior hippocampus. Error bars denote the standard error of the mean

Comparing the hippocampal volumes of memory athletes with matched controls, we found a trend for a main effect of group (*F*_(1,40.81)_ = 3.585, *p* = 0.065), this was further qualified by the interaction of group and anterior/posterior (*F*_(1,44)_ = 5.41, *p* = 0.025) and an interaction of group and hemisphere (*F*_(1,44)_ = 5, *p* = 0.03). Follow-up simple effect tests revealed an enlarged anterior hippocampus (MD 195.33, *p* = 0.016) but not posterior hippocampus (MD = − 39.97, *p* = 0.44). Additionally, we observed a main effect of hemisphere (*F*_(1,44)_ = 89.97, *p* < 0.001) with the right hippocampus (MD 111.42, *p* = 0.021) but not the left (MD 43.94, *p* = 0.289) being larger in memory athletes. The three-way interaction of group, anterior/posterior and hemisphere was not significant (*F*_(1,44)_ = 2.58, *p* = 0.115). From the covariates, only intracranial volume (*F*_(1,41)_ = 11.694, *p* = 0.001) had a significant effect. Age (*F*_(1,41)_ = 0.3, *p* = 0.581) and gender (*F*_(1,41)_ = 0.009, *p* = 0.932) did not show a significant effect. As both the right hippocampus and the anterior portion were enlarged in athletes, the right anterior hippocampus exhibited the largest group difference (Fig. 1).

Comparing the volume of the caudate nucleus between groups using a similar linear mixed model—only leaving out the anterior/posterior factor—we did not observe significant group differences: neither a main effect of group (*F*_(1,42.859)_ = 0.585, *p* = 0.449), nor of hemisphere (*F*_(1,43.928)_ = 0.312, *p* = 0.579), nor an interaction of group and hemisphere (*F*_(1,43.928)_ = 0.913, *p* = 0.345).

To test whether larger volumes of the hippocampus or the caudate nucleus would be beneficial for the memory athletes, we correlated the structural volumes—separately per structure—to the position in the world ranking. The right posterior hippocampus (*r*_(20)_ = 0.547, *p* = 0.008, Bonferroni corrected) and the right caudate nucleus (*r*_(20)_ = 0.5, *p* = 0.018, Bonferroni corrected) predicted the ranking. The association of the hippocampus was specific for the posterior part as indicated by a significantly stronger correlation compared to the left (*z* = 2.356, *p* = 0.018) and right (*z* = 2.501, *p* = 0.012) anterior hippocampus (see Table 1 for a full list of the correlations). In a control analysis, we recalculated all correlations with the world ranking using not only intracranial volume as a covariate but also age and gender. This did not change the significance of any result presented here.

**Table 1:**
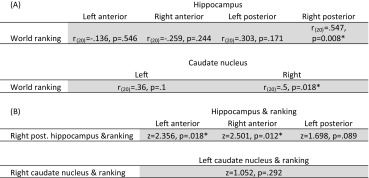
Association of the volumes of the hippocampus and caudate to the memory sports world ranking

(A) Correlations for all hippocampal and caudate nucleus structures with the world ranking position. Significance at the *p* < 0.05 level is indicated with a * after applying Bonferroni correction. (B) Comparing the correlation of the structures to the ranking reported above between the different structures (Steiger 1980). The values reported show whether the right posterior hippocampus and the right caudate nucleus show a significantly stronger correlation with the world ranking than the other structures

**Fig. 2:**
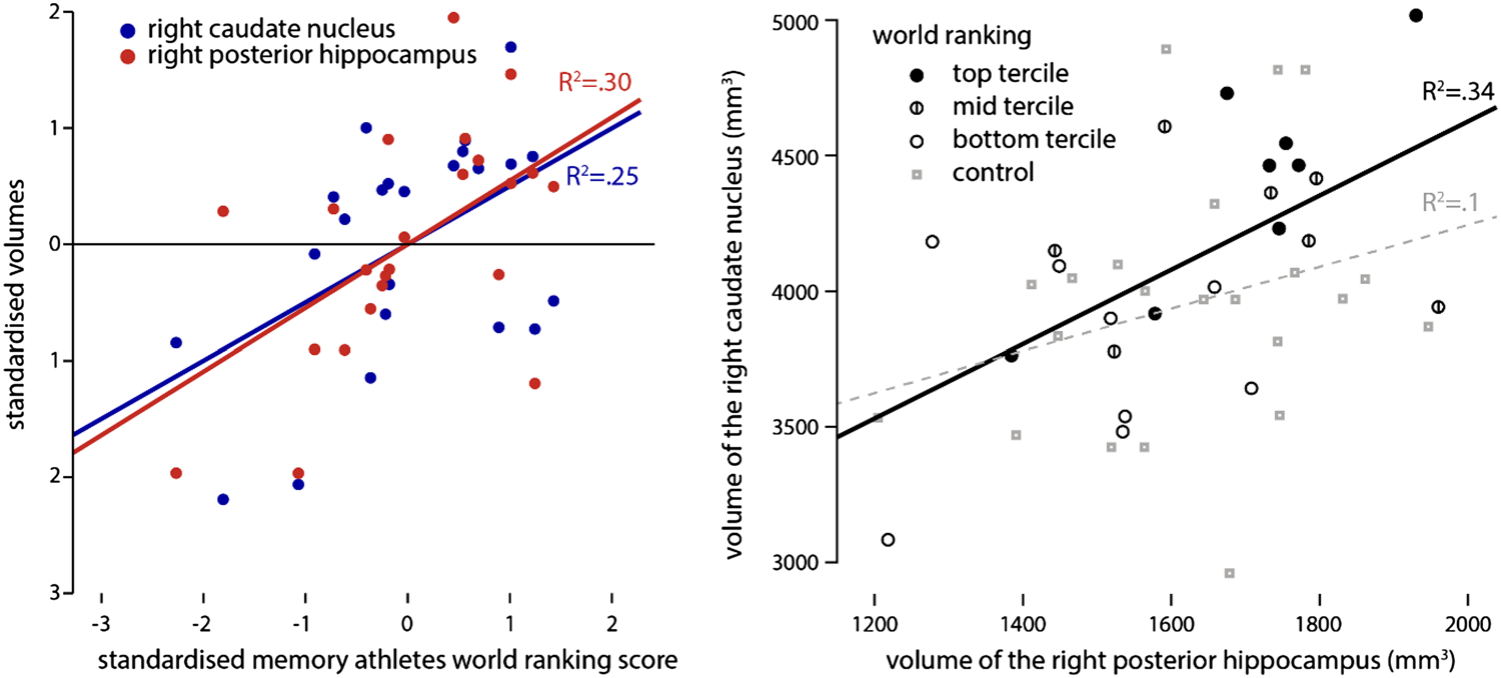
Volume of the right posterior hippocampus and the right caudate nucleus predicts the world ranking (left). Both the volume of the right posterior hippocampus (*r*(20) = 0.547, *p* = 0.008) and the right caudate nucleus (*r*_(20)_ = 0.5, *p* = 0.018) significantly predict the world ranking position of the memory athletes (right). Furthermore, we found a strong correlation between the volume of the right posterior hippocampus and the right caudate nucleus within the athletes (*r*_(20)_ = 0.633, *p* = 0.008). This correlation is significantly stronger compared to the control group (*z* = 2.2, *p* = 0.028). In which, the correlation seemed absent (*r*(20) = 0.05, *p* = 0.824). All correlations are corrected for intracranial volume

As both the right posterior hippocampus and right caudate nucleus correlated with the world ranking, we wanted to know whether they would in itself be strongly correlated or whether they are both independently linked to the world ranking. To test this, we correlated these volumes with each other in both the athlete and control group and compared them. We observed a strong correlation between the volumes in memory athletes (*r*_(20)_ = 0.633, *p* = 0.002), which was not detectable in the matched controls (*r*_(20)_ = 0.05, *p* = 0.824). By comparing them, we established that the memory athletes have a significantly larger association of those volumes than the matched controls (*z* = 2.2, *p* = 0.028). Taken together, the memory athletes exhibit a strong relation between the right posterior hippocampus and the right caudate nucleus volume; both of these volumes predict their ranking (Fig. 2).

### Functional connectivity

We found the strongest volumetric group difference in the right anterior hippocampus (Fig. 1). One may expect the biggest difference to have a strong relevance for the level of memory performance, however, rather than the right anterior hippocampus, it was the volume of the right posterior hippocampus and the right caudate nucleus that predict the world ranking (Fig. 2). Using the resting state data, we now wanted to test whether these two effects are functionally related, indicating a shared mechanism, or whether they are functionally unrelated, suggesting a different mechanism. To this end we calculated the functional connectivity (Pearson correlation) of the right anterior hippocampus with the right caudate nucleus and the right posterior hippocampus.

**Fig. 3:**
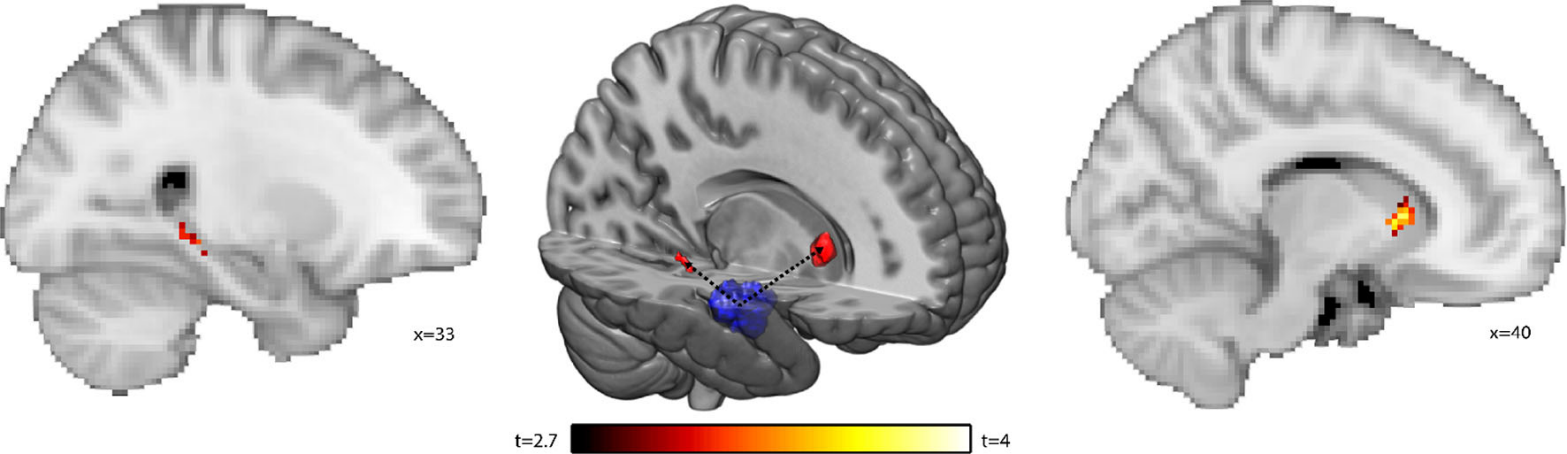
Seed-based functional connectivity analysis of the right anterior hippocampus (middle). We calculated the connectivity using the right anterior hippocampus as a seed (blue) to the right posterior hippocampus and the right caudate nucleus. The red areas demark voxels which connectivity to the right anterior hippocampus significantly (*p* < 0.05, tfce-corrected) predicts the world ranking of the memory athletes (left, right) the same results displayed in MNI space for illustration purposes

There were no group differences in functional connectivity of the right anterior hippocampus between groups (*p* > 0.05). Within the athletes, functional connectivity from right anterior hippocampus to both the right posterior hippocampus and right caudate nucleus predicted the world ranking position (all *p* < 0.05, small volume corrected; Fig. 3). In a control analysis, we verified that the right anterior hippocampus is significantly correlated to both the right posterior hippocampus and the right caudate nucleus (*p* < 0.001, tfce-corrected).

## Discussion

Comparing 23 of the world’s leading memory athletes with carefully matched controls, we observed enlarged hippocampal volumes, especially pronounced in the right anterior division. In contrast, volumes of the caudate nucleus volumes did not differ significantly from those of matched controls. The position in the memory sports world ranking was predicted by both the volume of the right caudate nucleus and the right posterior hippocampus. A second feature distinguishing the groups was that for memory athletes, the volumes of the right posterior hippocampus and the right caudate nucleus were more strongly correlated than in matched controls. Using resting state data, we observed an association between the structural group difference in the right anterior hippocampus and correlations with performance. Functional connectivity from the anterior hippocampus to both the right caudate nucleus and the right posterior hippocampus predicted the ranking.

We suggest that these results are best understood in the context of cooperative hippocampal–caudate nucleus interaction that may enable the superior performance seen in memory athletes. We focused on the caudate nucleus and the hippocampus, because both the ability to create simple stimulus response associations—supported by the caudate nucleus—and the utilisation of map-like representations—supported by the hippocampus—are essential aspects of the method of loci. A differential neural architecture regarding these structures that makes memory athletes more apt at utilising the method of loci might manifest itself in two ways. First, athletes might be characterized by enlarged pivotal brain structures. Second, they might utilise neural mechanisms not readily available to normal controls. For this reason, we compared our sample of memory athletes with matched controls, and complementary, we related the structural and functional variation we find in the sample of memory athletes to their position in the world ranking, thus identifying what makes certain memory athletes especially successful.

Three of our results provide evidence for the model that memory athletes utilise hippocampal–caudate in a cooperative fashion to enhance their ability to memorise information: volumes of the posterior hippocampus and caudate nucleus were associated with the world ranking; these two volumes are more strongly correlated with each other within the athletes compared to the matched controls. Resting state functional connectivity of the anterior hippocampus to both the posterior hippocampus and the caudate nucleus predicted the world ranking. Memory athletes with both a large posterior hippocampus and caudate nucleus were able of more impressive memory feats across different types of material. On top of that, the better athletes showed a stronger functional connectivity between those two regions and the anterior hippocampus, a region that showed the largest volumetric difference relative to matched controls. As memory athletes attribute their exceptional memory abilities to mnemonic strategies, such as the method of loci (Dresler et al. 2017; Dresler and Konrad 2013; Maguire et al. 2003), we propose that our findings reflect the degree to which the athlete’s neural architecture supports the use of mnemonic strategies by an optimised, cooperative utilisation of the caudate nucleus and hippocampus. It is important to note that the memory athletes excel in different memory domains, such as face memory, word list learning, memorising playing cards, not only in competitions but also in laboratory settings (Konrad 2014). However, differing from other forms of superior memory, such as highly superior autobiographic memory (LePort et al. 2016) or superior recognition abilities (Russell et al. 2009), the memory athletes are not intrinsically better at memorising, they need their techniques for their exceptional memory performance (Konrad 2014; Ramon et al. 2016). The method of loci in itself has been applied in diverse sets of context to facilitate memory (Worthen and Hunt 2011). The biggest advantage one would have to apply mnemonics in real life is when there are large amounts of information that have to be learned, especially when the material in itself is not very well structured (as for example a story is). However, to use the methods on the level of the athletes, a large amount of training will be necessary. Some of the memory athletes have told us that they used their mnemonics to learn medical terms or a new language rather quickly. Though even naïve participants can tremendously improve using the method of loci (Dresler et al. 2017) to learn word lists. Thus, if one is willing to practice the mnemonics and has to learn large sets of facts or associations by heart mnemonics seem like a good way of facilitating learning.

In the past, the debate of the interaction between the caudate nucleus and the hippocampal memory systems was focused on a competitive interaction (Poldrack and Packard 2003), with the systems competing for solving the task at hand (Packard and McGaugh 1996). The central evidence for competition that has been replicated multiple times by now is the following: before the rats solve a navigational task in which different task requirements can be fulfilled by either system, one of the relevant structures gets lesioned. Trivially, behaviour depending on this structure drops substantially. But importantly, behaviour that depends on the other structure is improved after the lesion. This increase suggests that the lesioned structure was competing for solving the task (Jacobson et al. 2012; McDonald and White 1994). Compared to the amount of work supporting the competitive notion, there is only preliminary evidence for cooperation of these systems: the hippocampus can compensate for dysfunction of the caudate nucleus during the early stages of Huntington’s disease; but providing even stronger support for cooperation was a functional interaction between the hippocampus and the right caudate nucleus in healthy controls facilitating route recognition (Voermans et al. 2004). However, beyond the competition vs. cooperation dichotomy there is also work that suggests parallel processing that not necessarily implies cooperation or competition (Doeller et al. 2008). Most of the evidence for competition of the memory systems comes from rather simple navigation paradigms in which there are only two choices; one indicating use of the stimulus response system, the other indicating a more spatial hippocampal strategy. However, with the method of loci combining aspects from stimulus-response learning—such as rapid navigation from one locus to the next in a fixed order—and aspects of hippocampal processing—such as scene construction (anterior hippocampus) and maintaining of a spatial representation of the route (posterior hippocampus)—a cooperation of those two systems seems optimal to produce exceptional memory performance. As these aspects utilised in the method of loci are quite complementary, we presume that the different systems can cooperate rather than interfere with each other as was shown in other navigational tasks (Packard and McGaugh 1996).

Extending this reasoning to our results suggests that the memory athletes show higher levels of cooperation between the hippocampus and caudate nucleus, thus facilitating the use of the method of loci.

One finding that links especially nicely to our results is that participants who focused stronger on a spatial strategy compared to a response-based strategy in a virtual navigation task showed increased grey matter density in the hippocampus while it was reduced in the caudate nucleus. Additionally, these densities were negatively correlated (Bohbot et al. 2007). This result is in line with the competition account: as the hippocampus and the caudate nucleus compete a high density of the hippocampus entails a relative lower density of the caudate nucleus and in turn there is an associated bias towards the hippocampal spatial strategy. In our memory athletes, we found the opposite pattern: the volumes of the right posterior hippocampus and right caudate nucleus were positively correlated; this correlation was significantly reduced and not apparent in matched controls (Fig. 2). If competition between memory systems leads to an inverse structural relation as described above, cooperation could lead to a positive association between structures. As for an example reported by Voermans et al. (2004), if the hippocampus is more dominant in a competing scenario it will suppress the caudate nucleus. In a cooperative scenario, it would support it. Whereas the matched controls do not utilise the two systems together frequently, the memory athletes do so, which goes hand in hand with a correlation between the structures involved. The structural consequences of this competition have recently been demonstrated by using video games as a model for spatial navigation (West et al. 2017). Players that relied on stimulus-response strategies showed a reduction in hippocampal grey matter, whereas players with a spatial strategy showed an increase.

The biggest volumetric difference in the memory athletes is the enlarged right anterior hippocampus. Since the work on taxi drivers’ navigational memory (Maguire et al. 2000; Woollett 2011), we know that the hippocampus remains plastic even after maturation. Extensive training in the method of loci could have similar neuroanatomical consequences for memory athletes as the acquisition of navigational memory in taxi drivers. However, since we do not have longitudinal data, we can only speculate whether enlarged hippocampi were a prerequisite or a consequence of the participants becoming world class memory athletes. For the taxi drivers, the hippocampal growth was linked to the acquisition of the complex street layout of London. As we lack a clear intervention in the memory athletes, we can only speculate about the differences in hippocampal volume. One facility that is central to the mnemonics utilised by the memory athletes is the ability to integrate information to enhance remembering it. During the method of loci, athletes have to transform the information they need to remember in a vivid image which is then associated with one of the route points of a very familiar environment. This function of integrating separate elements into a coherent visual scene has been linked to the anterior hippocampus (Zeidman et al. 2015; Zeidman and Maguire 2016).

The structural differences in the hippocampus and the association to the world ranking in the memory athletes was mostly right lateralised. For the caudate nucleus, results seemed stronger for the right hemisphere, however, they did not significantly differ. The right lateralisation for the hippocampus is in line with a substantial body of work showing the right hippocampus to more strongly implicated with spatial processing (Bohbot et al. 1998; Burgess et al. 2002; Kühn and Gallinat 2014; Postma et al. 2008). As the method of loci is a dominantly spatial one, it fits that the right side is more strongly implicated in the exceptional memory exhibited by the memory athletes we studied.

One central limitation of our study is that we did not investigate subdivisions of the caudate nucleus. From animal work, we know that there is spatial differentiation within the caudate nucleus in terms of cooperation and competition (Packard et al. 1989; Sabatino et al. 1992; McDonald and White 1993; Devan et al. 1999). Therefore, for future work it is important to use methods complementary to volumetry as we applied here. For example, voxel-based morphometry or shape analysis could help to dissociate cooperative from competitive sub-regions of the caudate nucleus. Another limitation is that we do not know how specific the cooperation of the caudate nucleus and the hippocampus is for the method of loci. Given how well video games might serve as a model for these spatial learning strategies (West et al. 2017), it might be interesting to have participants play a game that can best be performed if both strategies are integrated, as they are in the method of loci.

## Conclusion

We provide initial evidence that a cooperative interaction of the hippocampus and the caudate nucleus might enable world’s leading memory athletes to perform exceptional feats of memory. Volumes of the right posterior hippocampus and the right caudate nucleus were more strongly correlated within the group of athletes than in matched controls. The larger both structures were and the more strongly they were functionally coupled with the right anterior hippocampus, which was enlarged in athletes, the higher the rank the rank of the athlete.

## Electronic supplementary material

Below is the link to the electronic supplementary material.

Supplementary material 1 (DOCX 1454 kb)
