# Supplementary material for: Hippocampal–caudate nucleus interactions support exceptional memory performance
Source: Brain Struct Funct. 2017 Nov 14;223(3):1379–89. doi: 10.1007/s00429-017-1556-2 (PMC5869896; doi:10.1007/s00429-017-1556-2)
Supplement: Supplementary file 1 — Supplementary material 1 (DOCX 1454 kb) [file 429_2017_1556_MOESM1_ESM.docx]

# Supplemental Material

|  | memory athletes | matched controls |
| --- | --- | --- |
| n | 23 | 23 |
| males | 14 | 14 |
| age (years): mean ±SD | 27.8 ±8.6 | 28.1 ±8.1 |
| age (years): range | 19-51 | 20-53 |
| reasoning | 131.8 ±12.1 | 131.7 ±12.1 |
| memory | not tested | 103.5 ±25.5 |
| left-handers | 3 | 3 |
| smokers | 1 | 1 |

**Table T1. Related to Experimental Procedures**. Participant demographic details of the memory athletes and matched controls. Sample size, number of males, left-handers, smokers are given as absolute numbers; reasoning and memory abilities are given as mean IQ scores ± standard deviations.

|  | **Memory athletes** | **Matched controls** |
| --- | --- | --- |
| left anterior hippocampus | 1631.29 ± 295.17 | 1486.07 ± 226.33 |
| left posterior hippocampus | 1504.61 ± 222.92 | 1538.22 ± 172.72 |
| right anterior hippocampus | 1861.29 ± 370.05 | 1592.13 ± 268.62 |
| right posterior hippocampus | 1628.25 ± 190.12 | 1650.84 ± 194.95 |
| left caudate nucleus | 4101.86 ± 475.55 | 4029.13 ± 429.12 |
| right caudate nucleus | 4117.54 ± 449.48 | 3969.34 ± 472.55 |

**Table T2. Structural volumes**. Average volumes for the separate structures in mm^3^ and their standard deviation.

**
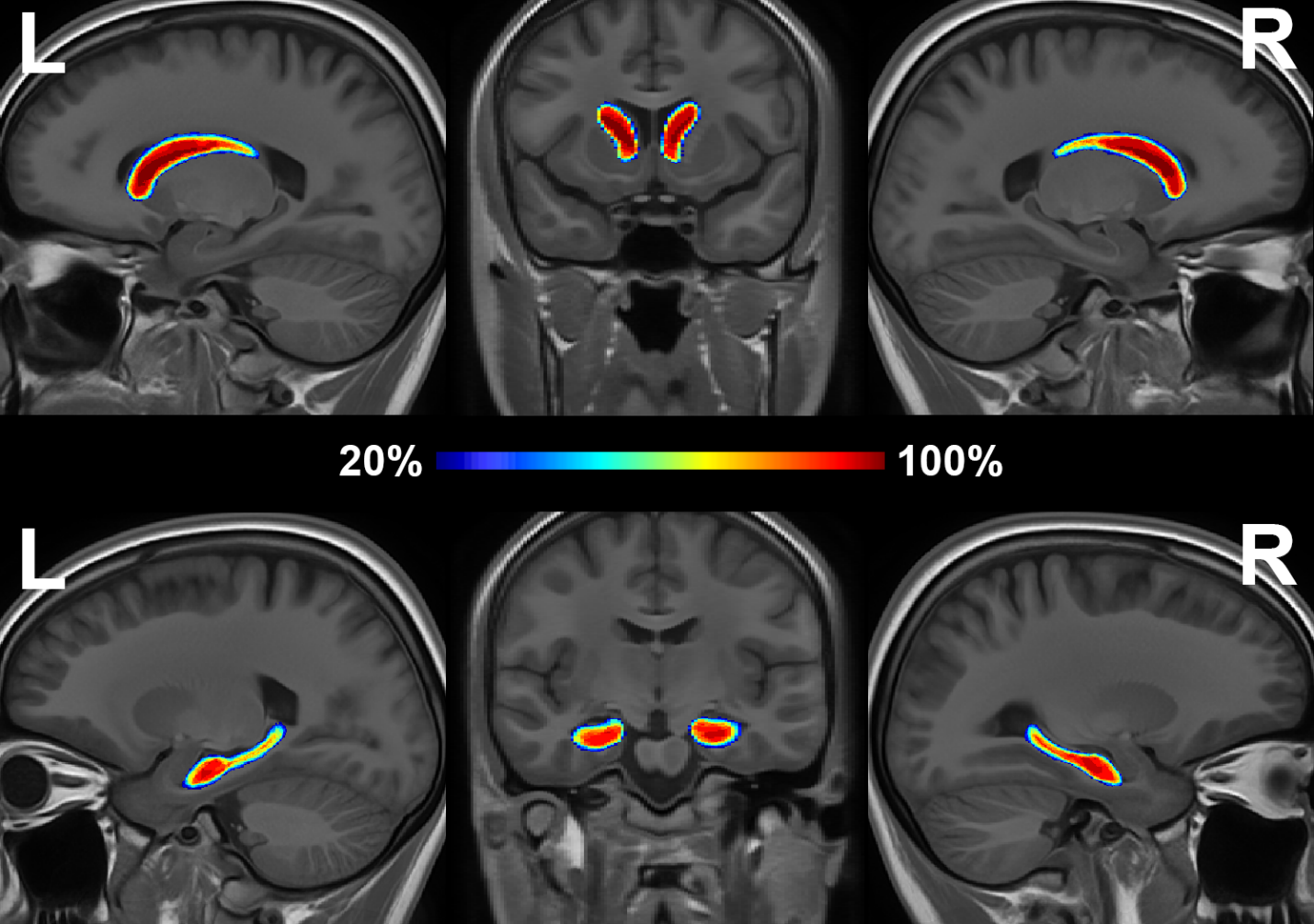
**

**Figure SF1. Illustration of the hippocampal and caudate segmentation.** Probability maps of the segmentations for the caudate nucleus (top) and the hippocampus (bottom) for the memory athletes. These depictions are only meant to illustrate the segmentations as all the volumetric statistics were extracted from the native space preventing registration errors affecting the volumetric estimates. Images were thresholded at 20%. All images here are depicted in study space that was generated using ANTs (see method section for details).

**
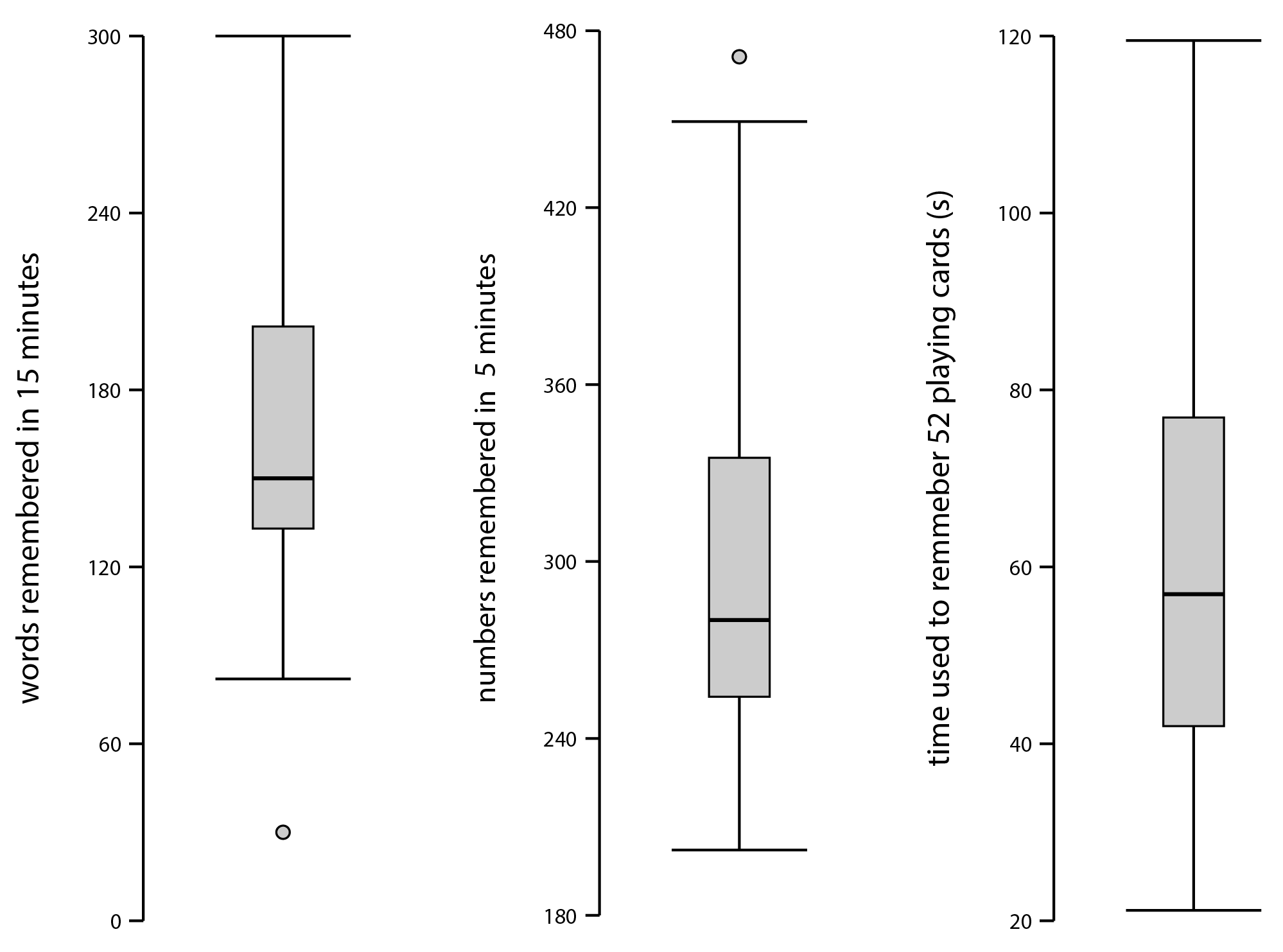
**

**Figure SF2. Performance of the memory athletes for different disciplines.** The memory athletes excel in a variety of disciplines. To illustrate this, we present here their records at the time of the study for three representative disciplines used in memory competition. In the words/numbers memory athletes get a list with random words or numbers and have to remember as many as they can within 15/5 minutes. The goal of the speed cards discipline is to remember a standard deck of 52 playing cards. The athletes receive a deck that is randomly shuffled. When they are done memorizing they give a signal and the time is stopped. The time only counts when the athlete is able to recall the order of the set of cards perfectly. For all these scores it is important to note that there are substantial deduction from the score if any error is made to prevent guessing. Thus, a lower score can be indicative of either a lower rate of correct items or a higher rate of incorrect items. The bold line depicts the median, the lower part of the box the 25% quantile, the upper part of the box the 75% quantile, the whiskers extend until the furthest data point that is still within the 1.5 interquantile range, dots outside the whiskers depict outliers.
